# Genome-Wide Analysis of the MADS-Box Gene Family in Maize: Gene Structure, Evolution, and Relationships

**DOI:** 10.3390/genes12121956

**Published:** 2021-12-07

**Authors:** Da Zhao, Zheng Chen, Lei Xu, Lijun Zhang, Quan Zou

**Affiliations:** 1 School of Applied Chemistry and Biological Technology, Shenzhen Polytechnic, Shenzhen 518055, China; zhaoda@webmail.hzau.edu.cn (D.Z.); chenzheng7@uestc.edu.cn (Z.C.); c7zlj@szpt.edu.cn (L.Z.); 2 School of Electronic and Communication Engineering, Shenzhen Polytechnic, Shenzhen 518055, China; 3 Institute of Fundamental and Frontier Sciences, University of Electronic Science and Technology of China, Chengdu 610054, China

**Keywords:** maize, gene family, MADS-box, transcription factor, phylogenetic analysis

## Abstract

The MADS-box gene family is one of the largest families in plants and plays an important roles in floral development. The MADS-box family includes the SRF-like domain and K-box domain. It is considered that the MADS-box gene family encodes a DNA-binding domain that is generally related to transcription factors, and plays important roles in regulating floral development. Our study identified 211 MADS-box protein sequences in the *Zea mays* proteome and renamed all the genes based on the gene annotations. All the 211 MADS-box protein sequences were coded by 98 expressed genes. Phylogenetic analysis of the MADS-box genes showed that all the family members were categorized into five subfamilies: MIKC-type, Mα, Mβ, Mγ, and Mδ. Gene duplications are regarded as products of several types of errors during the period of DNA replication and reconstruction; in our study all the 98 MADS-box genes contained 22 pairs of segmentally duplicated events which were distributed on 10 chromosomes. We compared expression data in different tissues from the female spikelet, silk, pericarp aleurone, ear primordium, leaf zone, vegetative meristem, internode, endosperm crown, mature pollen, embryo, root cortex, secondary root, germination kernels, primary root, root elongation zone, and root meristem. According to analysis of gene ontology pathways, we found a total of 41 pathways in which MADS-box genes in maize are involved. All the studies we conducted provided an overview of MADS-box gene family members in maize and showed multiple functions as transcription factors. The related research of MADS-box domains has provided the theoretical basis of MADS-box domains for agricultural applications.

## 1. Introduction

Maize (*Zea mays*) is an essential crop that is a staple food in many parts of the world, and the total production of maize has surpassed that of whole rice and wheat. The history of the agricultural selection of domesticated maize has spanned more than 10,000 years [1]. Maize is a staple food for people and fodder for animals; in addition, maize can also be widely used in energy and industry, such as in the production of ethyl alcohol, corn starch, and corn syrup [2]. Maize is an important model organism for genetic and genomic research [3]. Elie Dolgin completed the maize reference genome (B73) in 2009 and found that it contained a 2.3 billion base sequences and more than 32,000 protein-coding genes [2].

Transcription factors combined with specific DNA sequences are cis-acting elements that control promoters and enhancers. Transcription factors and target genes are combined through the DNA-binding area, a site that is the most conserved sequence in evolution. The MADS-box gene was first identified as a homologous gene controlling flower development that encodes a DNA-binding domain that is generally related to transcription factors [4]. The MADS-box domain (SRF-like and K-box like) functions in DNA-binding, protein dimerization, and nuclear localization [5]. MADS-box genes are widely expressed in almost all eukaryotes and some prokaryotes; many studies have been conducted in animals, plants, fungi, and other organisms [6]. MADS-box family genes also play essential roles in the plant life cycle, from embryo to gametophyte development [7]. MADS-box genes are crucial in regulating plant development, such as in female gametophytes, embryoid seed development, and fruit ripening [6,8].

Gramzow et al. divided MADS-box genes into two subtypes—SRF-like (Type I) and MEF2-like genes (Type II) [6]. The K-box domain has often been used to distinguish Type I and Type II genes. The K-box domain has approximately 70 amino acids and has been divided into three subtypes—K1, K2, and K3 [9]. Based on the MADS-box genes’ structure in *Arabidopsis thaliana*, Type I can be subdivided into four subgroups, Mα, Mβ, Mγ, and Mδ [10,11,12], while Type II genes can be subdivided into two subgroups, MIKC^C^ and MIKC* [6]. In addition, Type I genes consist of variable domains as well as SRF-like domains; in contrast, Type II genes consist of an intervening domain (I), a keratin-like domain (K), and a C-terminal domain (C) following MEF2-like domains [9]. The structural difference in I domains and K domains is features that distinguish MIKC^C^ and MIKC* [13]. The analysis of thousands of MADS-domain proteins has revealed that the MADS-box genes share the MIKC domain which enables protein combinatorial and cooperative multimerization [14].

MADS-box genes ZAG1 and ZAG2 were first identified as floral-development regulators in maize [15]. With the development of research, they were implicated in the absorption of nutrients in the root [16], starch biosynthesis [17], yield enhancement [18], and other processes [19]. Analyzing the evolution and function of MADS-box genes could help us understand the roles of the MADS-box gene family members in gene regulation interactive networks, which could reveal the origin, heredity, and evolution of species and provide a theoretical basis and technical support for variety breeding.

In this study, we analyzed MADS-box genes in maize. We fetched the MADS-box seed sequences for SRF-like and K-box domains (PF00319 and PF01486) from the protein family database (Pfam) [20]. A total of 211 protein sequences were identified, in which 176 sequences contained SRF-like domains and 181 sequences contained K-box domains. We analyzed the MADS-box genes in maize genome-wide. According to their evolutionary relationships, chromosome distribution, and family-member gene structures, the collinearity of MADS-box genes among three crops (rice, maize, and wheat) was analyzed. Based on transcription data from public databases, we explored the expression modules in different tissues and the development process of maize. We also outline the related research of MADS-box domains which could provide the theoretical basis of MADS-box domains for yield breeding in maize.

## 2. Materials and Methods

### 2.1. Identification and Classification of MADS-Box Proteins in Maize

The genomic sequence (Zea_mays.B73_RefGen_v4.dna.toplevel.fa), protein sequences (Zea_mays.B73_RefGen_v4.pep.all.fa) and annotation file (Zea_mays.B73_RefGen_v4.47.gff3) were downloaded from the Ensembl plants website (http://plants.ensembl.org/ (9 July 2020)). To identify all MADS-domain proteins in maize, a hidden Markov model (HMM) search was performed against the protein database of maize using the *SRF-TF* domain (PF00319) and the K-box domain (PF01486), which were downloaded from the Pfam protein family database (http://pfam.xfam.org/ (9 July 2020)) [20].

We used HMMR3.0 (http://hmmer.org/download.html (9 July 2020)) to search MADS-box genes from the maize genome with an e-value of 1e-10 [21]. All the MADS-box genes in maize whose structures were identified in SRF or K-box domains by SMART (http://smart.embl.de/ (9 July 2020)) were analyzed [22]. We used Clustalw for multiple protein sequence alignment to build a new hidden Markov model, which was used to extract protein sequences. All the candidate MADS-box genes were verified by Pfam and NCBI (National Center for Biotechnology Information). Furthermore, we analyzed the physicochemical properties, including the number of amino acids, molecular weight, isoelectric point, grand average of hydropathicity (GRAVY), and subcellular localization (Table S1). The number of amino acids, molecular weight, and isoelectric points were determined using a Perl script; GRAVY was calculated by the GRAVY calculator (http://www.gravy-calculator.de/ (26 June 2021)); and the subcellular localization was analyzed by BUSCA (http://busca.biocomp.unibo.it/ (26 June 2021)) [23].

### 2.2. Phylogenetic Analysis and Classification of the MADS Gene Family

To further understand the phylogenetic relationship of MADS-box gene family members in dicotyledons, we construct a phylogenetic tree with *Oryza sativa, Zea mays,* and *Triticum aestivum* L. A total of 655 protein sequences were identified, including 67 rice protein sequences, 211 maize protein sequences, and 377 wheat protein sequences. Muscle was used to carry out the alignment of multiple protein sequences [24]. The phylogenetic relationship was obtained by integrating the protein sequences using IQ-TREE software (multicore version 1.6.12) [25] with the best-fit model VT+F+R5 [26]. We assessed the phylogenetic relationships by ultrafast bootstrapping with 1000 replicates. Figtree was employed to visualize the results of phylogenetic relationship analysis (http://tree.bio.ed.ac.uk/software/figtree/ (13 July 2021)).

### 2.3. Chromosomal Distribution and Gene Structure Analysis of MADS-Box Genes in Zea mays

The genomic sequence (Zea_mays.B73_RefGen_v4.dna.toplevel.fa), protein sequences (Zea_mays.B73_RefGen_v4.pep.all.fa), and annotation file (Zea_mays.B73_RefGen_v4.47.gff3) were downloaded from the Ensembl website in the previous step. We used Tbtools to transform the gff3 file to a gtf file for further analysis. We extracted candidate MADS-box gene sequences and annotation files using a Perl script. The conserved motifs were blasted in the NCBI batch web conserved-domain tool (https://www.ncbi.nlm.nih.gov/Structure/bwrpsb/bwrpsb.cgi (14 January 2021)). We extracted the MADS-box gene sequence using a Perl script, including 2000 bp upstream of the CDS, to analyze types of cis-elements on the PlantCARE website (http://bioinformatics.psb.ugent.be/webtools/plantcare/html/ (20 May 2021)). The chromosomal distribution, cis-elements, and motif locations were visualized using Tbtools software (https://github.com/CJ-Chen/TBtools (22 January 2021)) [27].

### 2.4. Gene Duplication and Synteny Analysis of MADS-Box Genes in Zea mays

All MADS-box genes were mapped to the chromosomes of maize, and the physical location information was obtained from the genome sequence from annotation files of the maize genome (Zea_mays.B73_RefGen_v4.47.gff3) using a Perl script. Multiple collinear scanning toolkits (MCScanX) were used to analyze the gene replication events with default parameters. To study the synteny relationships between orthologous MADS genes of maize and other species, home linear-analysis maps were generated using Dual Synteny Plotter software (https://github.com/CJ-Chen/TBtools (26 May 2021)).

### 2.5. Expression and Regulation Relationships among MADS-Box Gene Family Members in Maize

The maize expression data of B73 (classical maize cultivar) throughout the growth period were acquired from the database Zeamap (http://www.zeamap.com/ (6 January 2021)) [28]. Each MADS-box gene was annotated and analyzed as a node in the regulation network utilizing the webserver STRING (https://string-db.org/ (7 January 2021)); we obtained the protein–protein interactions between MADS gene family members from public sources [29]. The interaction relationships were obtained by cystoscopy (https://cytoscape.org/ (7 January 2021)). The GO enrichment and KEGG pathways were analyzed using the cluego plugin integrated into Cystoscope [30].

## 3. Results

### 3.1. Identification of MADS-Box Gene Family Members in Maize

A total of 211 putative coding sequences corresponding to the MADS-box family were identified by searching the *Zea mays* genome with the SRF-TF domain (PF00319) and the K-box domain (PF01486). There were 176 SRF members and 185 K-box members with each domain, among which 117 members had both SRF and K-box domains. Among the 211 putative MADS-box proteins in maize, all the 211 MADS-box protein sequences were coded by 98 expressed genes. The protein numbers, molecular weights, and theoretical pIs ranged from 46 amino acids to 491 amino acids (5.4 kDa to 54.8 kDa and 4.12 to 11.84, respectively. Based on the results of GRAVY calculations, we found that most of the family members were hydrophilic proteins, while only a few of the members were hydrophobic proteins. The distribution of subcellular locations included the nucleus, chloroplast, extracellular space, and endomembrane system. Furthermore, we found that all hydrophobic proteins were related to chloroplast and endomembrane systems (Table S1).

### 3.2. Classification and Phylogenetic Analysis of the MADS-Box Gene Family Members

**Figure 1:**
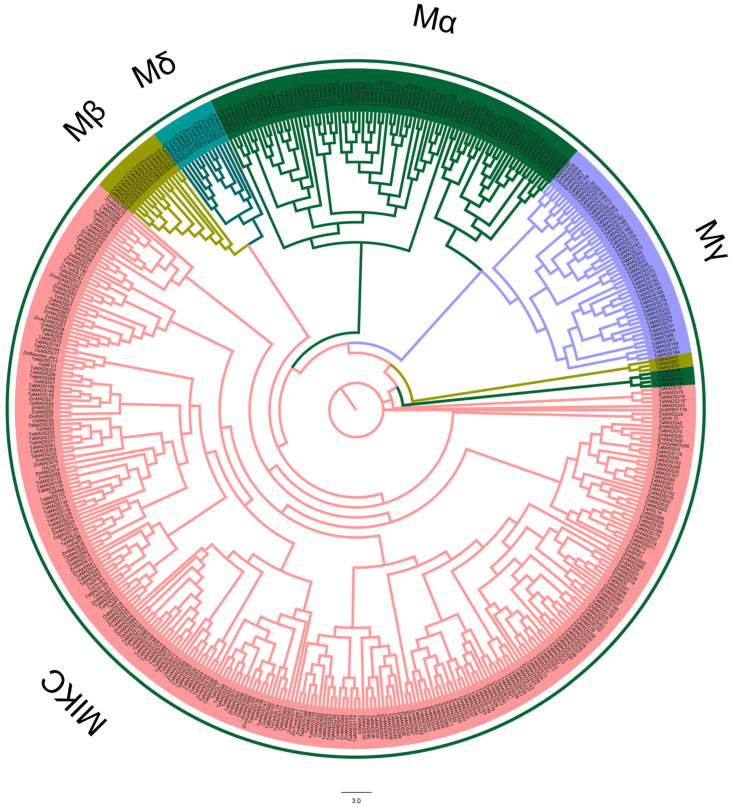
Phylogenetic tree of MADS-box protein sequences in *Oryza sativa*, *Zea mays*, and *Triticum aestivum* L. Phylogenetic analysis of 463 MADS-box protein sequences from MADS-box genes were divided into five subfamilies—MIKC-type, Mα, Mβ, Mγ, and Mδ.

In our study, a total of 67 rice protein sequences, 211 maize protein sequences, and 377 wheat protein sequences were identified by HMM analysis. All the 655 protein sequences were summarized into 463 groups by expressed genes. To construct the phylogenetic tree, we selected 463 protein sequences consisting of 60 rice protein sequences, 98 maize protein sequences, and 305 wheat protein sequences (Figure 1). Based on the formal study of MADS-box genes in *Arabidopsis*, a total of 108 genes were selected for classifying the MADS-box gene members [31]. From the phylogenetic tree, we found that the MADS-box genes were divided into five subfamilies—MIKC-type, Mα, Mβ, Mγ, and Mδ (Figure 1). 

### 3.3. Gene Structure and Motif Composition of the MADS-Box Gene Family

**Figure 2:**
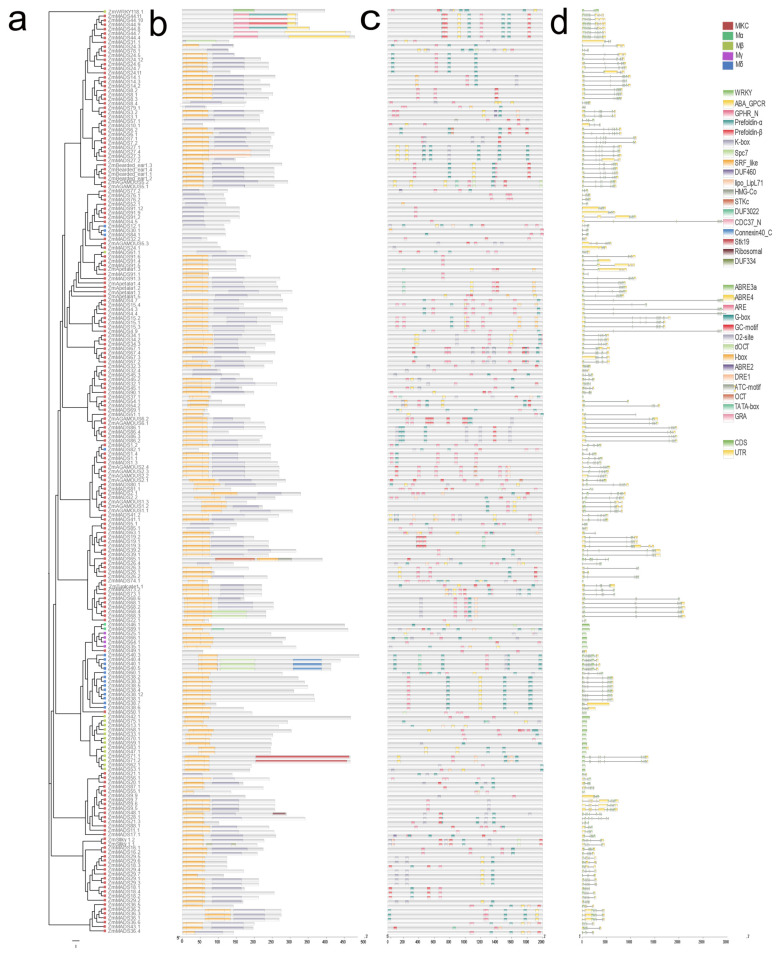
Phylogenetic relationships, gene structures, and conserved protein motifs of MADS-box genes from *Zea mays*. (**a**) The phylogenetic tree of 211 MADS-box proteins. (**b**) Motif composition of MADS-box proteins in maize; a total of 17 types of conserved domains were found. (**c**) Cis-element analysis of the MADS-box gene family. Different colors represent a total of 14 types of functional modules. (**d**) Gene structure of MADS-box genes. Black lines indicate introns, green boxes indicate CDS, and yellow boxes indicate UTR.

To further understand the distinct regions of proteins and motifs, we conducted an online analysis of the 211 putative MADS-box protein sequences in maize in the NCBI Conserved Domain Database. We constructed the phylogenetic tree of 211 MADS-box proteins in maize and annotated MIKC-type, Mα, Mβ, Mγ, and Mδ in different colors (Figure 2a). As shown in Figure 2b, we found a total of 17 types of conserved domains. In addition to SRF-like and K-box domains, which are the characteristic domains of MADS-box gene family members, ABA GPGR, WRKY, DUF3032, and Spc7 superdomains also play essential roles in MADS-box genes.

In addition, we selected 2000 bp of a CDS sequence promoter region and submitted it to PlantCare (http://bioinformatics.psb.ugent.be/webtools/plantcare/html/ (20 May 2021)) and summarized all the cis-elements into 14 types of functional modules (Figure 2c and Table S3). AREs, G-boxes, GC motifs, O2 sites, and ABRE3 exist widely in maize MADS-box family members. To further understand the composition of MADS-box genes in maize, we analyzed the domains in MADS-box genes and obtained the exon and intron structures from the GFF3 annotation file (Zea_mays.B73_RefGen_v4.47. gff3). The MADS-box genes were composed of several exons and introns, with significant differences (Figure 2d).

### 3.4. Gene Dupulacation, Chromosomal Distribution, and Synteny Analysis of MADS-Box Gene Family Members

**Figure 3:**
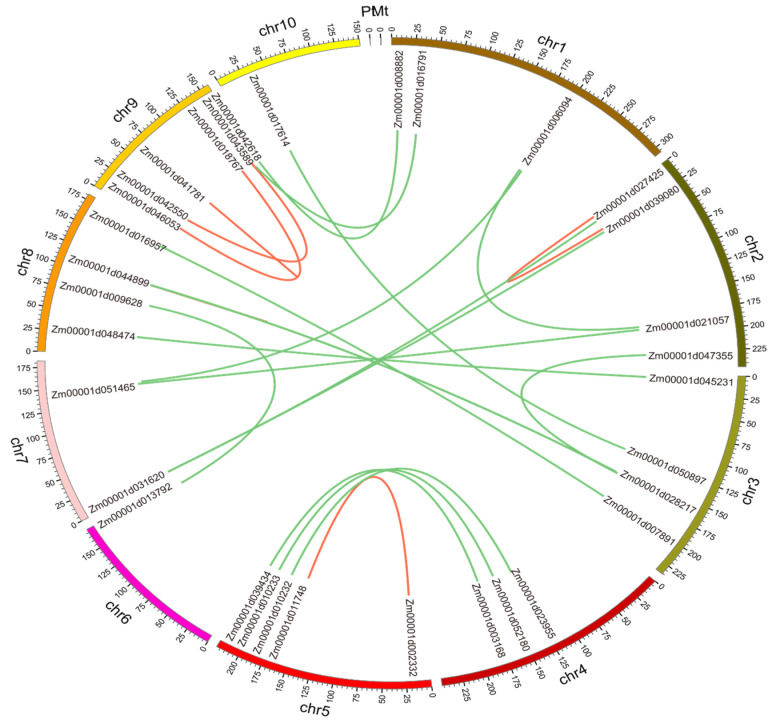
The intrachromosomal segmental duplication map of the MADS genes in maize. Colored lines inside the circle represent duplication inside the same chromosome (red) and between different chromosomes (green).

We used 98 MADS-box genes’ DNA sequences to analyze the concatenated duplication events in maize. We noticed that one gene might have two duplication events. If a gene had two duplicated events in another location, we would count it as two pairs. A total of 22 pairs of duplicated genes were distributed on 10 chromosomes (Figure 3). These results demonstrated that replication events were the fundamental driving force of MADS-box gene evolution. To further understand the evolutionary constraints acting on MADS-box gene family members, we calculated the Ka/Ks ratios of the MADS gene pairs. The orthologous MADS-box gene pairs had Ka/Ks < 1, suggesting that the maize MADS-box genes were under purifying selection pressure during evolution [32]. 

**Figure 4:**
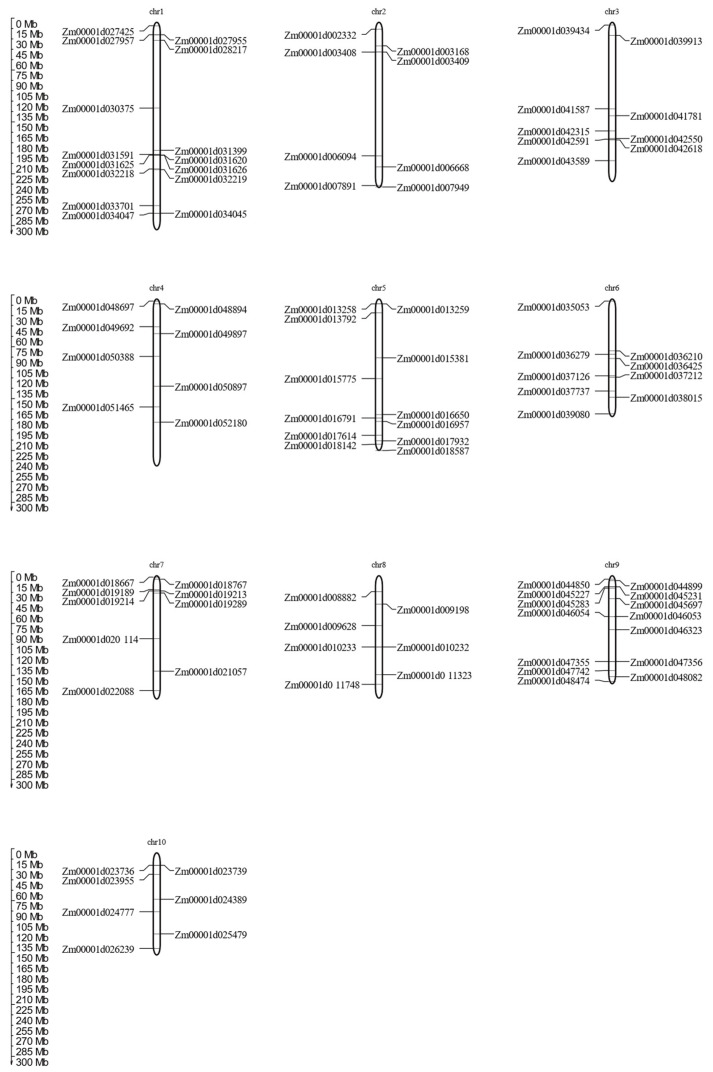
Chromosome distribution of the MADS-box genes in maize. The marks on the left represent the location of these genes on their chromosome.

The MADS-box gene family members were symmetrically distributed on 10 chromosomes of *Zea maize* linkage groups (Figure 4). Chromosome 1 had more MADS-box gene family members than the others. In contrast, chromosome 10 had the fewest members. The distribution of MADS-box gene members was significantly different between all 10 chromosomes. Considering the significant difference in MADS-box gene family members, genetic recombination and exchanges play important roles in the distribution of MADS-box genes.

### 3.5. Synteny Analysis of MADS-Box Gene Family Members in Maize and Other Species

**Figure 5:**
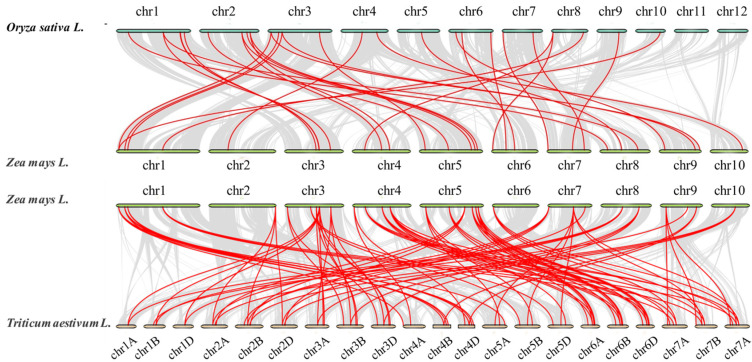
Synteny analysis of MADS-box family. The species are *Oryza sativa*, *Zea mays*, and *Triticum aestivum* L. Red lines are the syntenic MADS-box gene pairs for dicotyledon crop genomes. Gray lines are collinear blocks for dicotyledon crop genomes.

To further understand the phylogenetic mechanisms of MADS-box genes among *Zea mays*, *Oryza sativa*, and *Triticum aestivum* L., we analyzed the synteny relationships of these three important dicotyledonous crops (Figure 5). Gray lines represent the synteny relationships between two species and red lines represent the synteny relationships between two species in MADS-box gene family members. Based on the collinearity analysis of MADS-box genes between *Zea mays* and two other important crops (*Oryza sativa* and *Triticum aestivum* L.), we found that the MADS-box genes between *Zea mays* and *Triticum aestivum* L. have more homologous gene pairs with the MADS-box genes between *Oryza sativa* and *Triticum aestivum* L. The collinearity findings are listed in Supplementary Table S2.

### 3.6. Gene Expression in MADS-Box Gene Family Members

**Figure 6:**
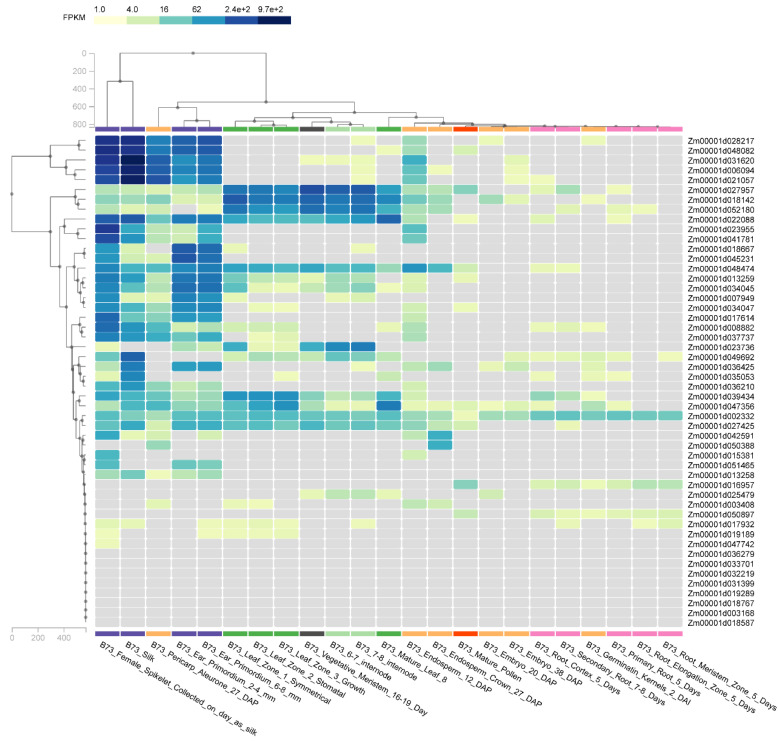
Expression of MADS-box genes in maize. Heat map of expression data in different tissues. The blue color represents higher expression levels and yellow represent lower expression levels.

We acquired the maize expression data of B73 (a classical maize cultivar) from the database Zeamap (http://www.zeamap.com/ (6 January 2021)) [26]. Comparing expression data in different tissues from the female spikelet, silk, pericarp aleurone, ear primordium, leaf zone, vegetative meristem, internode, endosperm crown, mature pollen, embryo, root cortex, secondary root, germinating kernels, primary root, root elongation zone, and root meristem, we constructed a heatmap of 98 genes which belong to MADS-box gene family in maize (Figure 6). We found that most genes had higher expression levels in the female spikelet, silk, pericarp aleurone, and ear primordium, while only a few were expressed in the ear primordium, leaf zone, vegetative meristem, internode, and endosperm crown. The female spikelet, silk, and pericarp aleurone at twenty-seventh day after pollination(DAP), the ear primordium at 2–4 mm and 6–8 mm, the leaf zone at 2 stomatal, the vegetative meristem at 16–19 days, 6–7 internodes, 7–8 internodes, and mature leaf 8, the endosperm crown at 27 days, the mature pollen and embryos at 38 DAP, the root cortex at 5 days, the secondary root at 7–8 days, the germinating kernels at second day after inoculation(DAI), the primary root at 5 days, the root elongation zone at 5 days, and the root meristem at 5 days (Figure 6, Supplementary Figure S1). In B73, we found 29 MADS-box genes with no expression. Subsequently, we took the 29 genes to analyze the expression in 82 other varieties; only two genes (Zm00001d031399 and Zm00001d036279) had no expression (Supplementary Figure S2).

### 3.7. Gene Regulation Networks: Review of the Potential Connections

**Figure 7:**
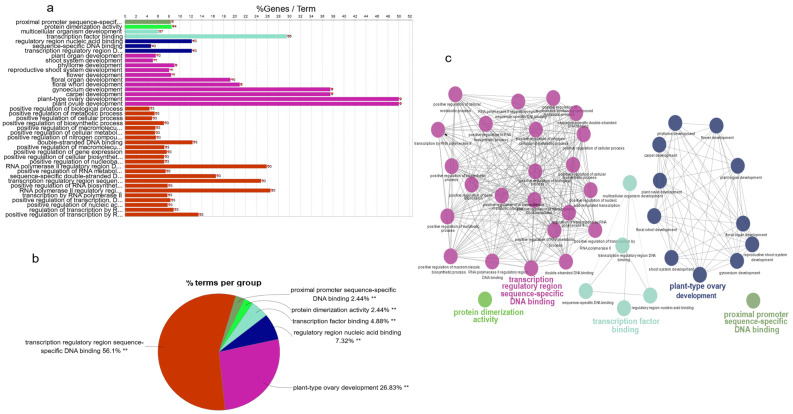
GO analysis, KEGG pathway analysis, and regulation networks of MADS-box genes in maize. (**a**) GO analysis of MADS-box genes in maize, a total of 41 pathways were found; (**b**) all 41 ontology pathways were classified into five groups; and (**c**) the interaction relationships of all 41 pathways in the five groups.

According to the analysis of gene ontology pathways, we found a total of 41 pathways in which MADS-box genes in maize were involved. Among them, sequence-specific DNA binding had the most associated genes (909), and plant ovule development and plant-type ovary development have the fewest associated genes (18). In contrast to MADS-box gene family members, protein dimerization had the most members (44), and proximal promoter sequence-specific DNA binding had the fewest members (3). By calculating the proportions of all the family members in pathway-associated genes, plant-type ovary development and plant ovule development accounted for the highest proportion (50%). In contrast, positive regulation of biological processes accounted for the lowest proportion (5%) (Figure 7a). All 41 ontology pathways could be classified into five groups—transcription regulatory region sequence-specific DNA binding (56.1%), plant-type ovary development (26.83%), transcription-factor binding (12.2%), protein dimerization (2.44%), and proximal promoter sequence-specific DNA binding (2.44%) (Figure 7b). Based on the PPI database, we identified the interaction relationships of all 41 pathways in the five groups (Figure 7c).

## 4. Discussion

The rapid progress of sequencing technology has made the study of genomes much more accessible [33]; studying gene family members’ functions and modes of action through the whole genome has proven to be an efficient method for exploring the interaction relationships in many types of gene families [34,35]. Many studies on the identification of gene family members in the whole genomes of different species, such as *Triticum aestivum* L. [31], *Populus trichocarpa* [36], *Brassica rapa* [37], *Brachypodium distachyon* [38], *Oryza sativa* [39], and *Arabidopsis* have been conducted [40].

Maize is an important crop and has the highest yield worldwide. MADS-box gene family members are essential transcription factors and play crucial roles in flowering and floral development [41]. Some studies also found that members of this family influenced female gametophytes, embryoid seed development, and fruit ripening [8,42]. We surveyed MADS-box gene family members’ functions that might be able to help us to breed and produce yield improvements in maize [43,44].

Previous work identified 75 genes in maize and divided them into 11 subfamilies, 7 duplication pairs, and 14 motifs; the expression of MADS-box genes in maize comes from mixed tissues and organs [42,43]. In contrast, our study identified 67 protein sequences in *Oryza sativa*, 211 protein sequences in *Zea mays*, and 377 protein sequences in *Triticum aestivum* L. for analyzing MADS-box gene family members. Furthermore, we studied the conserved motifs of 211 protein sequences in maize (Figure 2b) and found 17 types of conserved domains, in addition to SRF-like and K-box domains, which were the characteristic domains of MADS-box gene family members. HMG-CoA (β-hydroxy β-methylglutaryl-CoA) is a metabolic intermediate in the metabolism of branched-chain amino acids, which consist of leucine, isoleucine, and valine. [45,46]. ABA GPCRs are abscisic acid receptors that are GPCR-type G proteins related to elaborate receptor–effector signaling networks [47]. WRKY is one of the most important transcription factors in plants and appears to be involved in pathogen defense, senescence, and trichome ontogeny [48]. Prefoldin is a molecular chaperone involved in protein folding and synthesis [49]. Spc7 is an essential kinetochore protein that plays a crucial role in kinetochore-microtubule interactions [50]. The diversity of MADS-box gene family member motifs suggests that MADS-box genes may have more potential functions. We also identified 14 types of cis-regulatory elements in the MADS-box family members (Figure 2c). The I-box, which consists of GATAA, is an essential binding sequence of light-regulated sequences in plants [51]. ABREs play an essential role in response to ABA to affect osmotic stress and drought stress tolerance in plants [52,53]. GC motifs have been identified in response to waterlogging tolerance during cell wall modification in plants [54]. As “G-box” elements, they are involved in linalool biosynthesis during floral development [55]. The analysis of cis-regulatory elements has indicated that the promotor region is probably related to maize development and tolerance. All the MADS-box genes are composed of several exons and introns, with significant differences (Figure 2d). The diversity of cis-elements and motifs is proposed to have different types of regulatory mechanisms and functions. Most of the structural domains within *Zea mays* MADS-box genes have conserved motifs, and many studies in other plant species have suggested similar results [56,57]. These findings demonstrate that MADS-box genes in plants are highly conserved. However, some studies found that Type I MADS-box genes, especially Mα have a faster birth and death rate in maize [58]. 

Combining genome information analysis of rice, corn, and wheat, we know that their total numbers of genes are approximately 39,045 [59], 38,987 [2], and 107,891 [60], respectively, and the ratios of family members in maize are higher than those in rice and wheat. Perhaps this is related to the more frequent behavior of transposons in maize. The mechanisms involved in forming the gene family were considered to be gene duplication, relocation, divergence [61], and concerted evolution [62]. Gene duplications are regarded as products of several types of errors during DNA replication and reconstruction. Tandem replication, segmental replication, and genome duplication, rearrangement, and expansion also play essential roles in functional gene diversity [63]. We annotated MADS-box genes in maize from Ensembl plants; some related genes had been fine-mapped or cloned. Bearded-ear (*bde*) encodes transcription factors that affects FM determinacy, organ ontogeny, and sex determination [64,65]. Apetala1 in *Arabidopsis* controls floral-organ identity and inflorescence architecture [66], which has a homologous gene, *zap1*, in maize [15]. The *Arabidopsis* gene AGAMOUS1 was isolated as zag1 in maize [67], and mutants of *zag1* formed extra carpels and silks [68]. Tunicate1 (TU1) was detected in developing maize inflorescences [69] and has been proposed to play roles in floral development and sex determination [70,71]. In GO and KEGG pathway analyses, we explored the potential functions of MADS-box gene family members in maize. Through analysis of expression data, we found the MADS-box genes are functional in the ontogeny of the female spikelet, stigma, aleurone layer and panicle primordium; all 41 ontology pathways could be classified into five groups—transcription regulatory region sequence-specific DNA binding, plant-type ovary development, transcription factor binding, protein dimerization, and proximal promoter sequence-specific DNA binding. Go and KEGG analysis indicated that MADS-box genes play multiple roles in plant development as transcription factors. Related research on MADS-box domains has provided the theoretical basis of MADS-box domains for agricultural applications. The potential functions need to be verified and explored by more experiments in subsequent studies.

## Data Availability

Data is contained within the article or Supplementary Material.

## Supplementary Materials

The following are available online at https://www.mdpi.com/article/10.3390/genes12121956/s1, Figure S1: Expression of MADS-box genes in maize (48 genes); Figure S2: Expression_of_MADS-box genes in multiple maize cultivars (no expression genes in B73); Table S1: Details of MADS-box gene family members in maize; Table S2: One-to-one orthologous relationships; Table S3: Details of the cis-acting elements for MADS-box gene family members in maize.

## References

[1] Benz B.F. (2001). Archaeological evidence of teosinte domestication from Guila Naquitz, Oaxaca. Proc. Natl. Acad. Sci. USA.

[2] Dolgin E. (2009). Maize genome mapped. Nature.

[3] Lawrence C.J., Dong Q., Polacco M.L., Seigfried T.E., Brendel V. (2004). MaizeGDB, the community database for maize genetics and genomics. Nucleic Acids Res..

[4] Schwarz-Sommer Z., Huijser P., Nacken W., Saedler H., Sommer H. (1990). Genetic Control of Flower Development by Homeotic Genes in Antirrhinum majus. Science.

[5] Treisman R. (1995). DNA-binding proteins. Inside the MADS box. Nature.

[6] Gramzow L., Theissen G. (2010). A hitchhiker’s guide to the MADS world of plants. Genome Biol..

[7] Smaczniak C., Immink R.G., Angenent G.C., Kaufmann K. (2012). Developmental and evolutionary diversity of plant MADS-domain factors: Insights from recent studies. Development.

[8] Alvarez-Buylla E.R., Liljegren S.J., Pelaz S., Gold S.E., Burgeff C., Ditta G.S., Vergara-Silva F., Yanofsky M.F. (2008). MADS-box gene evolution beyond flowers: Expression in pollen, endosperm, guard cells, roots and trichomes. Plant J..

[9] Kaufmann K., Melzer R., Theissen G. (2005). MIKC-type MADS-domain proteins: Structural modularity, protein interactions and network evolution in land plants. Gene.

[10] Parenicova L., de Folter S., Kieffer M., Horner D.S., Favalli C., Busscher J., Cook H.E., Ingram R.M., Kater M.M., Davies B. (2003). Molecular and phylogenetic analyses of the complete MADS-box transcription factor family in *Arabidopsis*: New openings to the MADS world. Plant Cell.

[11] Nam J., Kim J., Lee S., An G., Ma H., Nei M. (2004). Type I MADS-box genes have experienced faster birth-and-death evolution than type II MADS-box genes in angiosperms. Proc. Natl. Acad. Sci. USA.

[12] Arora R., Agarwal P., Ray S., Singh A.K., Singh V.P., Tyagi A.K., Kapoor S. (2007). MADS-box gene family in rice: Genome-wide identification, organization and expression profiling during reproductive development and stress. BMC Genom..

[13] Henschel K., Kofuji R., Hasebe M., Saedler H., Munster T., Theissen G. (2002). Two ancient classes of MIKC-type MADS-box genes are present in the moss Physcomitrella patens. Mol. Biol. Evol..

[14] Theißen G., Gramzow L. (2016). Structure and Evolution of Plant MADS Domain Transcription Factors. Plant Transcription Factors.

[15] Mena M., Mandel M.A., Lerner D.R., Yanofsky M.F., Schmidt R.J. (1995). A characterization of the MADS-box gene family in maize. Plant J..

[16] Liu Y., Jia Z., Li X., Wang Z., Chen F., Mi G., Forde B., Takahashi H., Yuan L. (2020). Involvement of a truncated MADS-box transcription factor ZmTMM1 in root nitrate foraging. J. Exp. Bot..

[17] Dong Q., Wang F., Kong J., Xu Q., Li T., Chen L., Chen H., Jiang H., Li C., Cheng B. (2019). Functional analysis of ZmMADS1a reveals its role in regulating starch biosynthesis in maize endosperm. Sci. Rep..

[18] Wu J., Lawit S.J., Weers B., Sun J., Mongar N., Van Hemert J., Melo R., Meng X., Rupe M., Clapp J. (2019). Overexpression of zmm28 increases maize grain yield in the field. Proc. Natl. Acad. Sci. USA.

[19] El-Esawi M.A., Alayafi A.A. (2019). Overexpression of Rice Rab7 Gene Improves Drought and Heat Tolerance and Increases Grain Yield in Rice (*Oryza sativa* L.). Genes.

[20] El-Gebali S., Mistry J., Bateman A., Eddy S.R., Luciani A., Potter S.C., Qureshi M., Richardson L.J., Salazar G.A., Smart A. (2019). The Pfam protein families database in 2019. Nucleic Acids Res..

[21] Mistry J., Finn R.D., Eddy S.R., Bateman A., Punta M. (2013). Challenges in homology search: HMMER3 and convergent evolution of coiled-coil regions. Nucleic Acids Res..

[22] Letunic I., Doerks T., Bork P. (2012). SMART 7: Recent updates to the protein domain annotation resource. Nucleic Acids Res..

[23] Savojardo C., Martelli P.L., Fariselli P., Profiti G., Casadio R. (2018). BUSCA: An integrative web server to predict subcellular localization of proteins. Nucleic Acids Res..

[24] Edgar R.C. (2004). MUSCLE: Multiple sequence alignment with high accuracy and high throughput. Nucleic Acids Res..

[25] Nguyen L.T., Schmidt H.A., von Haeseler A., Minh B.Q. (2015). IQ-TREE: A fast and effective stochastic algorithm for estimating maximum-likelihood phylogenies. Mol. Biol. Evol..

[26] Kalyaanamoorthy S., Minh B.Q., Wong T.K.F., von Haeseler A., Jermiin L.S. (2017). ModelFinder: Fast model selection for accurate phylogenetic estimates. Nat. Methods.

[27] Chen C., Chen H., Zhang Y., Thomas H.R., Frank M.H., He Y., Xia R. (2020). TBtools: An Integrative Toolkit Developed for Interactive Analyses of Big Biological Data. Mol. Plant.

[28] Gui S., Yang L., Li J., Luo J., Xu X., Yuan J., Chen L., Li W., Yang X., Wu S. (2020). ZEAMAP, a Comprehensive Database Adapted to the Maize Multi-Omics Era. iScience.

[29] Szklarczyk D., Gable A.L., Lyon D., Junge A., Wyder S., Huerta-Cepas J., Simonovic M., Doncheva N.T., Morris J.H., Bork P. (2019). STRING v11: Protein-protein association networks with increased coverage, supporting functional discovery in genome-wide experimental datasets. Nucleic Acids Res..

[30] Bindea G., Mlecnik B., Hackl H., Charoentong P., Tosolini M., Kirilovsky A., Fridman W.H., Pages F., Trajanoski Z., Galon J. (2009). ClueGO: A Cytoscape plug-in to decipher functionally grouped gene ontology and pathway annotation networks. Bioinformatics.

[31] Ma J., Yang Y., Luo W., Yang C., Ding P., Liu Y., Qiao L., Chang Z., Geng H., Wang P. (2017). Genome-wide identification and analysis of the MADS-box gene family in bread wheat (*Triticum aestivum* L.). PLoS ONE.

[32] Hurst L.D. (2002). The Ka/Ks ratio: Diagnosing the form of sequence evolution. Trends Genet..

[33] Adams C.I.M., Knapp M., Gemmell N.J., Jeunen G.J., Bunce M., Lamare M.D., Taylor H.R. (2019). Beyond Biodiversity: Can Environmental DNA (eDNA) Cut It as a Population Genetics Tool?. Genes.

[34] Kingan S.B., Heaton H., Cudini J., Lambert C.C., Baybayan P., Galvin B.D., Durbin R., Korlach J., Lawniczak M.K.N. (2019). A High-Quality De novo Genome Assembly from a Single Mosquito Using PacBio Sequencing. Genes.

[35] Chen Z., Shen Z., Zhao D., Xu L., Zhang L., Zou Q. (2020). Genome-Wide Analysis of LysM-Containing Gene Family in Wheat: Structural and Phylogenetic Analysis during Development and Defense. Genes.

[36] Leseberg C.H., Li A., Kang H., Duvall M., Mao L. (2006). Genome-wide analysis of the MADS-box gene family in Populus trichocarpa. Gene.

[37] Duan W., Song X., Liu T., Huang Z., Ren J., Hou X., Li Y. (2015). Genome-wide analysis of the MADS-box gene family in Brassica rapa (Chinese cabbage). Mol. Genet. Genom..

[38] Wei B., Zhang R.Z., Guo J.J., Liu D.M., Li A.L., Fan R.C., Mao L., Zhang X.Q. (2014). Genome-wide analysis of the MADS-box gene family in *Brachypodium distachyon*. PLoS ONE.

[39] Tang Y., Wang J., Bao X., Wu Q., Yang T., Li H., Wang W., Zhang Y., Bai N., Guan Y. (2020). Genome-wide analysis of *Jatropha curcas* MADS-box gene family and functional characterization of the JcMADS40 gene in transgenic rice. BMC Genom..

[40] Zheng Y., Ren N., Wang H., Stromberg A.J., Perry S.E. (2009). Global Identification of Targets of the *Arabidopsis* MADS Domain Protein AGAMOUS-Like15. Plant Cell.

[41] Gramzow L., Ritz M.S., Theissen G. (2010). On the origin of MADS-domain transcription factors. Trends Genet..

[42] Adamczyk B.J., Fernandez D.E. (2009). MIKC* MADS domain heterodimers are required for pollen maturation and tube growth in *Arabidopsis*. Plant Physiol..

[43] Motorin Y., Helm M. (2019). Methods for RNA Modification Mapping Using Deep Sequencing: Established and New Emerging Technologies. Genes.

[44] Schilling S., Pan S., Kennedy A., Melzer R. (2018). MADS-box genes and crop domestication: The jack of all traits. J. Exp. Bot..

[45] Haines B.E., Steussy C.N., Stauffacher C.V., Wiest O. (2012). Molecular modeling of the reaction pathway and hydride transfer reactions of HMG-CoA reductase. Biochemistry.

[46] Rodwell V.W., Nordstrom J.L., Mitschelen J.J. (1976). Regulation of HMG-CoA reductase. Adv. Lipid Res..

[47] Pandey S., Nelson D.C., Assmann S.M. (2009). Two Novel GPCR-Type G Proteins Are Abscisic Acid Receptors in *Arabidopsis*. Cell.

[48] Eulgem T., Rushton P.J., Robatzek S., Somssich I.E. (2000). The WRKY superfamily of plant transcription factors. Trends Plant Sci..

[49] Sahlan M., Zako T., Yohda M. (2018). Prefoldin, a jellyfish-like molecular chaperone: Functional cooperation with a group II chaperonin and beyond. Biophys. Rev..

[50] Kerres A., Vietmeier-Decker C., Ortiz J., Karig I., Beuter C., Hegemann J., Lechner J., Fleig U. (2004). The fission yeast kinetochore component Spc7 associates with the EB1 family member Mal3 and is required for kinetochore-spindle association. Mol. Biol. Cell.

[51] Terzaghi W.B., Cashmore A.R. (1995). Light-Regulated Transcription. Annu. Rev. Plant Physiol. Plant Mol. Biol..

[52] Yoshida T., Fujita Y., Sayama H., Kidokoro S., Maruyama K., Mizoi J., Shinozaki K., Yamaguchi-Shinozaki K. (2010). AREB1, AREB2, and ABF3 are master transcription factors that cooperatively regulate ABRE-dependent ABA signaling involved in drought stress tolerance and require ABA for full activation. Plant J..

[53] Fujita Y., Yoshida T., Yamaguchi-Shinozaki K. (2013). Pivotal role of the AREB/ABF-SnRK2 pathway in ABRE-mediated transcription in response to osmotic stress in plants. Physiol. Plant.

[54] Arora K., Panda K.K., Mittal S., Mallikarjuna M.G., Thirunavukkarasu N. (2017). In Silico Characterization and Functional Validation of Cell Wall Modification Genes Imparting Waterlogging Tolerance in Maize. Bioinform. Biol. Insights.

[55] Yu Z., Zhang G., da Silva J.A.T., Zhao C., Duan J. (2021). The methyl jasmonate-responsive transcription factor DobHLH4 promotes DoTPS10, which is involved in linalool biosynthesis in *Dendrobium officinale* during floral development. Plant Sci..

[56] Shu Y., Yu D., Wang D., Guo D., Guo C. (2013). Genome-wide survey and expression analysis of the MADS-box gene family in soybean. Mol. Biol. Rep..

[57] Liu M., Fu Q., Ma Z., Sun W., Huang L., Wu Q., Tang Z., Bu T., Li C., Chen H. (2019). Genome-wide investigation of the MADS gene family and dehulling genes in tartary buckwheat (*Fagopyrum tataricum*). Planta.

[58] Zhao Y., Li X., Chen W., Peng X., Cheng X., Zhu S., Cheng B. (2010). Whole-genome survey and characterization of MADS-box gene family in maize and sorghum. Plant Cell Tissue Organ Cult. (PCTOC).

[59] Ouyang S., Zhu W., Hamilton J., Lin H., Campbell M., Childs K., Thibaud-Nissen F., Malek R.L., Lee Y., Zheng L. (2007). The TIGR Rice Genome Annotation Resource: Improvements and new features. Nucleic Acids Res..

[60] Appels R., Eversole K., Stein N., Feuillet C., Keller B., Rogers J., Pozniak C.J., Choulet F., Distelfeld A., Poland J. (2018). Shifting the limits in wheat research and breeding using a fully annotated reference genome. Science.

[61] Ohta T. (2008). Gene Families: Multigene Families and Superfamilies. Encyclopedia of Life Sciences.

[62] Ohta T. (2010). Gene conversion and evolution of gene families: An overview. Genes.

[63] Zhang S., Xu R., Luo X., Jiang Z., Shu H. (2013). Genome-wide identification and expression analysis of MAPK and MAPKK gene family in *Malus domestica*. Gene.

[64] Mendes-Moreira P., Alves M.L., Satovic Z., Dos Santos J.P., Santos J.N., Souza J.C., Pego S.E., Hallauer A.R., Vaz Patto M.C. (2015). Genetic Architecture of Ear Fasciation in Maize (*Zea mays*) under QTL Scrutiny. PLoS ONE.

[65] Thompson B.E., Bartling L., Whipple C., Hall D.H., Sakai H., Schmidt R., Hake S. (2009). bearded-ear encodes a MADS box transcription factor critical for maize floral development. Plant Cell.

[66] Bowman J.L., Alvarez J., Weigel D., Meyerowitz E.M., Smyth D.R. (1993). Control of flower development in *Arabidopsis thaliana* by APETALA1 and interacting genes. Development.

[67] Mena M., Ambrose B.A., Meeley R.B., Briggs S.P., Yanofsky M.F., Schmidt R.J. (1996). Diversification of C-function activity in maize flower development. Science.

[68] Laudencia-Chingcuanco D., Hake S. (2002). The indeterminate floral apex1 gene regulates meristem determinacy and identity in the maize inflorescence. Development.

[69] Han J.J., Jackson D., Martienssen R. (2012). Pod corn is caused by rearrangement at the Tunicate1 locus. Plant Cell.

[70] Collins G.N. (1917). Hybrids of *Zea Tunicata* and *Zea Ramosa*. Proc. Natl. Acad. Sci. USA.

[71] Langdale J.A., Irish E.E., Nelson T.M. (1994). Action of the Tunicate locus on maize floral development. Dev. Genet..

